# Current practice and innovations in diagnosing perianal fistulizing Crohn’s disease (pfCD): a narrative review

**DOI:** 10.1007/s10151-025-03122-6

**Published:** 2025-04-15

**Authors:** E. Anand, T. Pelly, S. Joshi, E. Shakweh, L. N. Hanna, A. Hart, P. Tozer, P. Lung

**Affiliations:** 1 St Mark’s The National Bowel Hospital, Central Middlesex, Acton Lane, London, UK; 2 Imperial College London, London, UK

**Keywords:** Diagnosis, Radiology, Fistula, Perianal Crohn’s disease

## Abstract

Perianal fistulizing Crohn’s disease (pfCD) represents a severe manifestation of Crohn’s disease (CD) that often leads to significant morbidity. Clinical examination alone of perianal fistulae is unlikely to be sufficient in the context of complex pfCD, as patients are likely to have complex disease and are more likely to experience complications, treatment failure, and recurrent disease. Furthermore, the relapsing–remitting nature of Crohn’s disease and our limited understanding of the pathogenesis of this potentially destructive disease necessitate regular examination and radiological assessment, often in the form of magnetic resonance imaging (MRI). Recent advancements in diagnostic techniques have enhanced the accuracy and timeliness of pfCD diagnosis, facilitating better patient outcomes. A growing appreciation of isolated perianal Crohn’s disease has prompted a recent attempt to develop consensus recommendations on diagnosing and treating this group of patients who would previously not have been offered CD medications. This narrative review aims to summarize current practice and the latest developments in the diagnosis of pfCD, highlighting:

1. Clinical examination and assessment tools
2. Current imaging practices
3. Innovations in imaging and biomarkers
4. The diagnosis of isolated perianal Crohn’s disease.

## Introduction

Perianal fistulizing Crohn’s disease (pfCD) is a particularly challenging subtype of CD, characterized by the development of abnormal epithelial connections between the anorectal canal and perianal skin [1]. A recent meta-analysis has suggested that approximately 1 in 5 patients with CD will experience perianal disease at some point during their lifetime [2], with 1 in 4 experiencing complications from perianal fistulae within the first two decades after their diagnosis [3, 4]. These fistulae can cause significant discomfort, infection, and complications that reduce patients’ quality of life, often leading to feelings of embarrassment [5, 6]. In the pre-biologic era, a study at St Mark’s Hospital highlighted the difficulty in treating complex as opposed to simple perianal fistulae, noting a high recurrence rate upon therapy discontinuation [7].

Early and precise diagnosis is therefore crucial, as undertreated disease is more likely to drive a greater symptom burden and reduction in quality of life [1]. Around two-thirds of patients undergo perianal surgery during their disease course, with around 6% requiring major surgery (colectomy, defunctioning -ostomy, proctectomy) [2]. Traditional diagnostic methods, including clinical evaluation with gentle finger pressure [8], are limited by the failure to detect deeper disease such as abscesses, extensions, and cavities. MRI has greater sensitivity for the detection of these complicated features that, if missed, are a frequent cause of treatment failure [9]. Evidence from the PISA II trial suggests that stopping treatment prematurely on the basis of clinical healing alone is a predictor of treatment failure and disease recurrence [10]. We know that radiological healing lags behind clinical healing, and therefore effective diagnosis and assessment of this lifelong condition requires regular imaging [11, 12]. Indeed, radiological healing is increasingly used as an endpoint in clinical trials of pfCD [13], although the precise definition of a radiologically improved and completely radiologically healed fistulae remains a point of controversy and is the subject of ongoing work by the Treatment Optimisation and Classification (TOpClass) Consortium of experts in perianal Crohn’s disease [14, 15]. Recent technological and methodological advancements offer promise of more accurate and comprehensive diagnostic approaches and are the subject of this narrative review.

## Clinical examination

### Clinical history and examination

Perianal fistulae can be the initial manifestation of CD in around 10% of patients [16]. Thorough history-taking is crucial, focusing on symptoms such as anal pain during defecation, perianal itching, bleeding, and purulent discharge—all of which are common in patients with a history of drained abscesses [17, 18]. Importantly, a family history of IBD, extraintestinal manifestations, or unexplained luminal symptoms of CD should prompt consideration of endoscopic evaluation for luminal disease, along with detailed imaging of complex fistulae indicative of pfCD. It is important to consider differential diagnoses for pfCD, which include traumatic lesions, hidradenitis suppurativa, tuberculosis, HIV infection, lymphogranuloma venereum, perianal actinomycosis, and post-rectal dermoid inclusion cyst, among others [17]. Clinicians should also be aware of the concept of isolated perianal Crohn’s disease (ipCD), in which a Crohn’s fistula exists in the absence of evidence of luminal disease. A novel expert consensus process has sought to define diagnosis and treatment in this group [19]. The diagnosis of a fistula in pfCD can be made clinically or on imaging. According to European Crohn’s and Colitis Organisation (ECCO)–European Society for Gastrointestinal and Abdominal Radiology (ESGAR) guidelines, MRI is recommended as the first-line diagnostic modality for perianal fistulizing Crohn’s disease (pfCD) [20]. The diagnosis of CD can be made on colonoscopy, assessment, or biopsy. This comprehensive approach ensures accurate diagnosis and aids in the effective management of pfCD.

### Classification

The classification of pfCD has evolved significantly over the years, with various systems being developed to improve diagnostic accuracy and treatment planning [21, 22]. This is addressed in a companion article in detail. From a diagnostic viewpoint, a fistula MRI, endoscopic assessment of the rectum, and clinical assessment are all important to determine fistula class. Table 1 presents the TOpClass consortium’s treatment and diagnostic recommendations for each category of perianal disease [22].

**Table 1:** Summary of recommendations for classes of perianal Crohn’s disease

TOpclass group	Class 1: minimal disease	Class 2a: chronic symptomatic fistulae suitable for repair	Class 2b: chronic symptomatic fistulae for symptom control	Class 2c-i: rapidly progressive disease	Class 2c-ii: gradually debilitating disease	Class 3: severe disease with exhausted perineum	Class 4a: post-proctectomy sinus/wound suitable for repair	Class 4b: post-proctectomy sinus/wound suitable for symptom control
Medical therapy	Establishing or continuing IBD therapies Individualized consideration of the need to escalate medical therapy	Ensure medical therapy optimized prior to repair	Consider escalation or switch in medical therapy	Prompt initiation of advanced medical therapy Proactive drug monitoring (TDM) and escalation of therapy as appropriate	Consider escalation or switch in medical therapy	Consider continuing advanced medical therapies post-proctectomy for perineal disease protection*	Consider optimizing or restarting advanced medical therapies to reduce perineal inflammation, particularly if perineal granulomata detected Consider hyperbaric oxygen therapy to support sinus/wound healing	
Setons	No role for routine seton insertion	Before repair: Preparatory seton usually recommended	Seton drainage generally advisable, although can be removed if deemed safe and impacting quality of life	Essential to optimize drainage and control disease	Consider long-term seton placement to reduce symptoms and improve quality	N/A	N/A	N/A
Surgical therapy	N/A	Surgical options include advancement flap, LIFT, and other SPPs for definitive repair Avoid fistula plugs/glue	Symptom control and rationalization: reduce symptoms by reducing the volume and inflammatory burden of fistula Rationalize the fistula to downstage to 2a** Consider drainage, setons, VAAFT, and other SPPs to close portions of fistula tracts	Serial EUAs to ensure adequate drainage Early referral to high-volume fistula centre Early consideration of stoma formation	Perform EUAs as required to ensure ongoing optimized drainage and rationalization of fistula complex as per class 2b Discuss the option of stoma formation	Proctectomy should be discussed and considered with patient	Surgical repair options include flap repair, cleft closure, etc. Drainage of presacral and pelvic collections, with consideration of excision of mesorectum, may support repair attempts	Optimize drainage and rationalize sinus complex Consider debriding sinus/wound and utilizing VAAFT and curettage
Recommended diagnostics and imaging	MRI is not routinely recommended for monitoring unless there is a change in symptoms	Before repair: Recent MRI Endoscopy to confirm absence of proctitis Clinical assessment for anal stricture and florid perianal disease After repair: Assess MRI response (e.g., at 6–12 months)	Serial MRIs to assess response to medical/surgical rationalization New MRI recommended if symptoms escalate EUAs may be needed to assess as well as for treatment	Serial MRIs to assess response to medical/surgical rationalization New MRI recommended if symptoms escalate EUAs may be needed to assess as well as for treatment Luminal assessment to determine optimal site of defunctioning stoma	Serial MRIs to assess response to medical/surgical rationalization New MRI recommended if symptoms escalate EUAs may be needed to assess as well as for treatment Luminal assessment to determine optimal site of defunctioning stoma	Serial MRIs to assess response to medical/surgical rationalization New MRI recommended if symptoms escalate EUAs may be needed to assess as well as for treatment Luminal assessment to determine optimal extent of resection	Before repair: Preoperative MRI for surgical planning and to identify complications (collections, connection to residual bowel, osteomyelitis, or malignancy) After repair: Assess MRI response (e.g., at 6–12 months)	Before repair: Preoperative MRI for surgical planning and to identify complications (collections, connection to residual bowel, osteomyelitis, or malignancy) After repair: Assess MRI response (e.g., at 6–12 months)

(Adapted from Hanna et al., 2024: Perianal Fistulizing Crohn’s Disease: Utilizing the TOpClass Classification in Clinical Practice to Provide Targeted Individualized Care) [22]

*SPPs*  sphincter-preserving procedures, e.g., fistula laser closure and VAAFT

Joint IBD–surgical care is recommended for complex cases

*Very limited supportive evidence

**Improvement in fistula morphology or associated perianal/rectal disease that would make the patient suitable for repair (i.e., transition to class 2a)

### Examination under anesthesia

Examination under anesthesia (EUA) is a critical tool in the diagnosis and management of pfCD, particularly in complex disease that cannot be assessed adequately in the clinic room with inspection and digital rectal examination (DRE). ECCO guidelines consider EUA to be the gold standard for evaluating fistula anatomy, especially when performed by an experienced colorectal surgeon [23]. It is particularly valuable in cases where imaging modalities such as MRI are contraindicated or unavailable [23, 24], but is also indicated in combination with imaging to obtain complete accuracy in assessment of fistula anatomy.

**Fig. 1:**
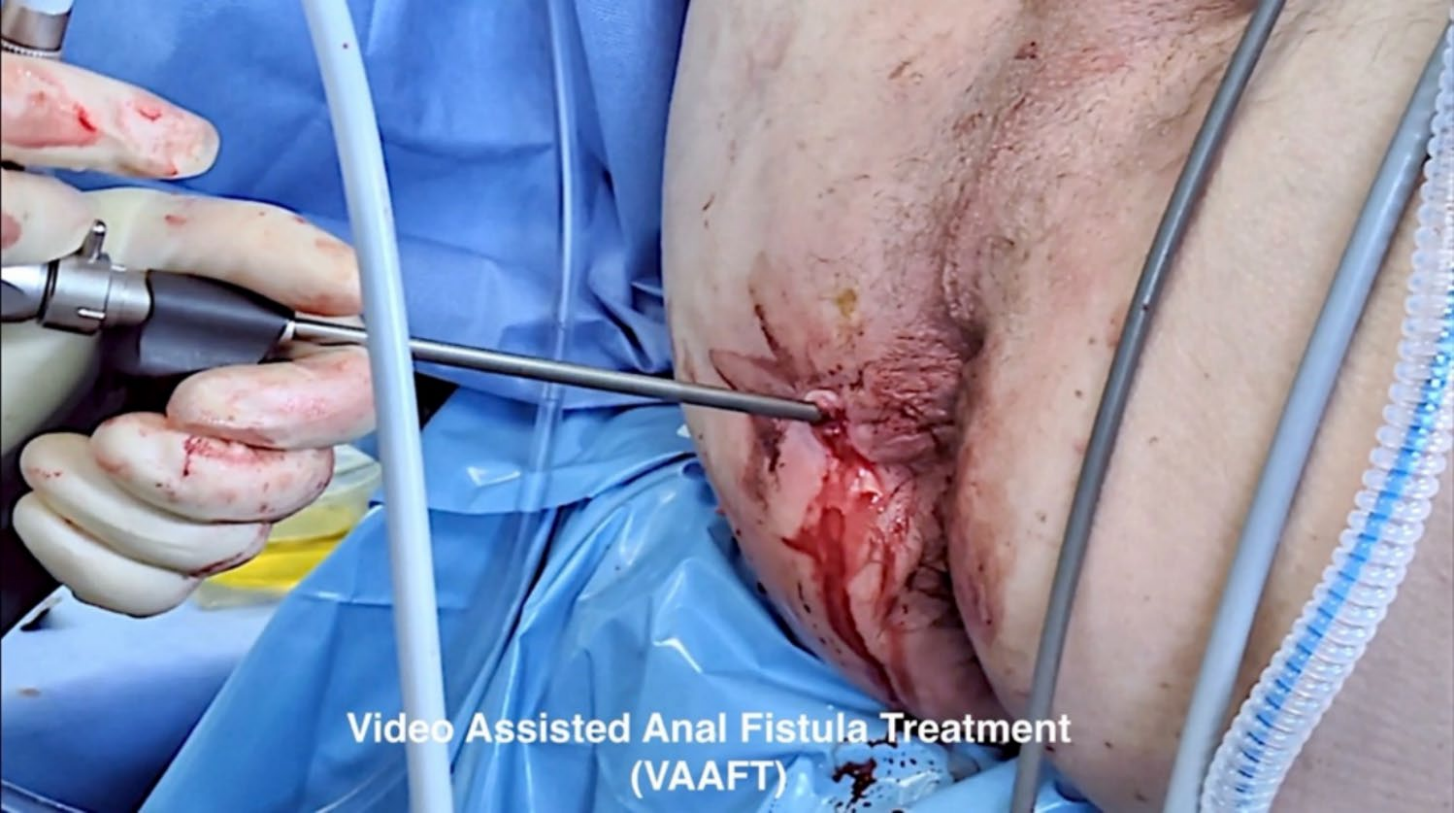
Video-assisted anal fistula treatment (VAAFT) (diagnostic and therapeutic)

The technique for EUA involves careful external examination, often with the patient in the lithotomy position, followed by digital examination, internal visual inspection, and palpation. Various probes may be used to explore fistulae, with adjuncts such as dilute hydrogen peroxide sometimes injected to better identify internal openings or complex tracks [25, 26]. This approach allows for the simultaneous therapeutic intervention, such as drainage of abscesses or placement of setons, often necessary in these cases [27, 28]. Newer minimally invasive tools, such as video-assisted anal fistula treatment (VAAFT), provide both diagnostic and therapeutic advantages [29, 30]. They enhance the detection of secondary tracts and extensions that routine EUA may overlook, while also allowing ablative cauterization of the fistula tract and precise identification of the internal opening (IO). This approach supports both rationalization and curative sphincter-preserving procedures when combined with IO closure. However, the use of VAAFT is limited by the requirement for a fistula tract wide (and straight) enough to allow passage of a 4.7-mm rigid scope (Fig. 1).

Despite its utility, EUA is not without drawbacks. Studies have shown that EUA may misclassify perianal fistulae in about 10% of patients, potentially leading to suboptimal therapeutic outcomes [31]. Therefore, European Society of Coloproctology (ESCP) guidelines recommend that EUA should not be the sole diagnostic tool in complex fistula cases, advocating for its use in conjunction with MRI or EAUS to achieve greater diagnostic accuracy [25, 32–34]. Overall, while EUA is a valuable procedure, its optimal use lies in being part of a multimodal diagnostic and therapeutic strategy.

### Endoscopy

Endoscopy is essential in the diagnosis and investigation of pfCD. Ileocolonoscopy with histology confirming luminal inflammation supports a diagnosis of pfCD and should be performed in patients with unexplained GI symptoms, complex perianal fistulae, or IBD risk factors [23, 35]. The presence of proctitis, detected through proctosigmoidoscopy, is significant as it has been consistently associated with poor fistula healing and a higher likelihood of proctectomy [7]. For patients in whom there is clinical suspicion of CD and ileocolonoscopy is unremarkable, small bowel evaluation should be considered. There are a number of modalities available to assess the small bowel radiologically with comparable sensitivities at detecting luminal inflammation, including CT enterography (CTE), MR enterography (MRE), and intestinal ultrasound (IUS). Choice of imaging depends on patient factors (such as age and tolerance to oral or intravenous contrast media) and availability of local resources and expertise. An alternative assessment tool is small bowel capsule endoscopy (SBCE); however, it is important to consider the suitability of this in patients with suspected small bowel stenosis or prior small bowel resections, where there is a risk of capsule retention [20]. SBCE has the highest diagnostic yield for the detection of proximal small bowel CD [36], whereas an imaging modality is more appropriate for disease mapping where the diagnosis has already been made.

## Current imaging practices

### MRI

**Fig. 2:**
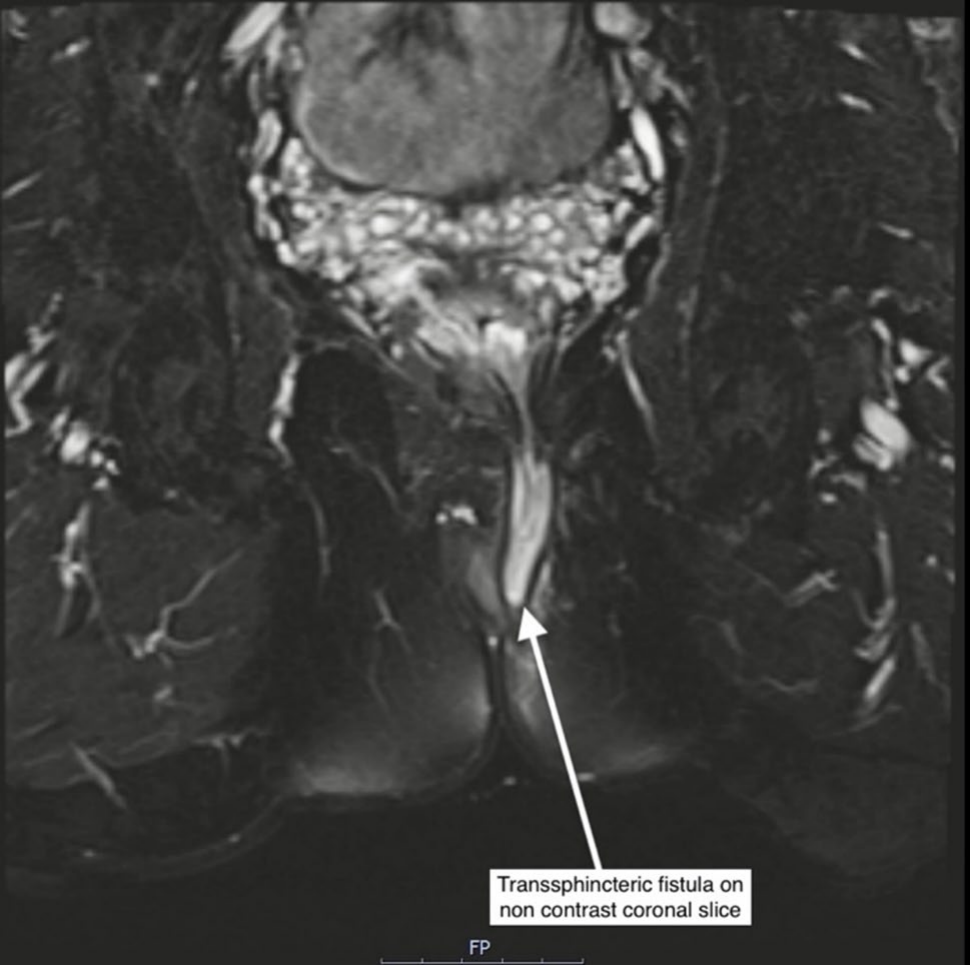
Transsphincteric fistula on noncontrast MRI coronal slice

Magnetic resonance imaging (MRI) is essential in diagnosing and managing pfCD, especially in complex cases. ECCO–ESGAR guidelines recommend MRI as the reference standard for evaluating perianal fistulae owing to its high diagnostic accuracy and ability to produce detailed images without ionizing radiation in often young patients undergoing serial imaging [1, 23, 35, 37, 38]. MRI boasts a sensitivity of 97% for detecting complex fistulae, surpassing clinical examination and being comparable to endoanal ultrasound (EAUS) [39]. Its specificity ranges from 76% to 100%, making it reliable for identifying fistula tracts and related structures [26, 37]. MRI is accurate in 90% of cases, compared with 81% with EAUS and 61% with EUA [40]. Combining MRI or EAUS with EUA further enhances both specificity and sensitivity [23] (Fig. 2), (Table 2).

**Table 2:** Summary of imaging modalities used in perianal Crohn’s disease

Modality	Strengths	Limitations	Notes
MRI	– Reference standard for pfCD diagnosis (ECCO–ESGAR)	– Can misclassify fistulae	– T2-weighted imaging essential
	– High sensitivity (97%) and specificity (76–100%)	– Not always available	– Gadolinium-enhanced T1-weighted imaging optional
	– Maps fistula tracts in multiple planes	– Radiological healing lags behind clinical remission (up to 12 months)	– First-line recommendation for assessment and ongoing surveillance
	– Can distinguish between active and fibrotic tracts		
	– Useful for treatment monitoring		
Endoanal ultrasound (EAUS)	– High spatial resolution, useful for assessing sphincter complex	– Operator-dependent results	– More useful for intersphincteric fistulae
	– Hydrogen peroxide contrast enhances visualization	– Narrow field of view	
	– 3D EAUS can identify Crohn’s ultrasound fistula sign (CUFS)	– Uncomfortable in acutely inflamed patients	
	–Alternative for patients intolerant to MRI (e.g., claustrophobia, metalwork)		
Transperineal ultrasound (TPUS)	– Alternative if rectal stenosis or pain prevents EAUS	– Operator-dependent results	– Complementary to MRI in specific cases
	– Alternative for patients intolerant to MRI (e.g., claustrophobia, metalwork)	– Lower sensitivity for deep fistulae	
		– Less commonly used	
CT and fistulography	– Useful in acute settings for abscess detection	– Ionizing radiation exposure	– Not recommended for routine use
	– Alternative for patients intolerant to MRI (e.g., claustrophobia, metalwork)	– Poor differentiation between fistulae and pelvic muscles	

**Fig. 3:**
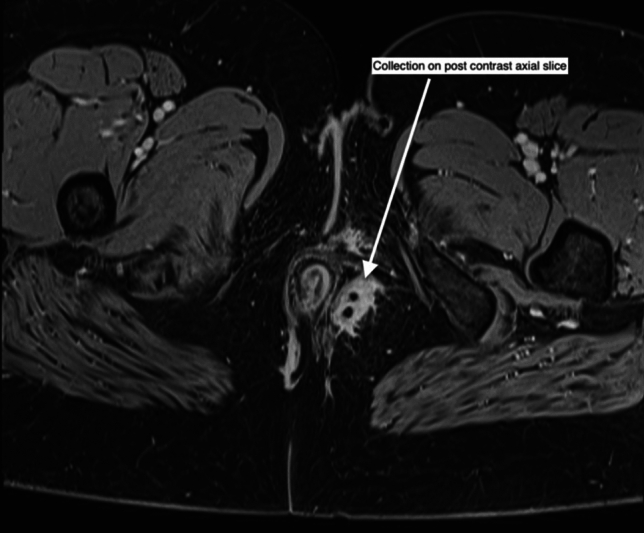
Collection on postcontrast MRI axial slice

MRI’s ability to clearly distinguish soft tissues and visualize structures in coronal and sagittal planes makes it highly effective for mapping fistula tracts and differentiating between patent, fibrotic, or mixed tracts [41]. For pfCD, MRI should at least include T2-weighted imaging, with optional gadolinium-enhanced T1-weighted sequences to distinguish between fluid, pus, or granulation tissue within fistula tracts [1, 35, 42] (Fig. 3). However, MRI has limitations; it may occasionally misclassify fistulae and might not be available in all settings [34]

**Fig. 4:**
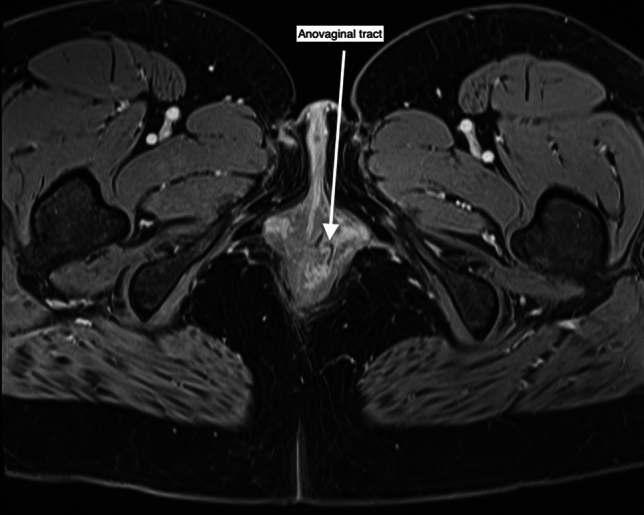
Anovaginal fistula on postcontrast MRI axial slice

Post-treatment MRI monitoring is used to evaluate the progress of complex pfCD following medical and surgical treatments. There is a significant correlation between MRI-assessed disease activity and clinical outcomes [43]. It is important to recognize that radiological healing on MRI can lag behind clinical remission by up to 12 months, underscoring the need for ongoing imaging follow-up even after clinical symptoms improve [11, 17]. Thus, MRI serves not only as a diagnostic tool but also as a key element in the continued management and treatment planning for pfCD (Fig. 4).

### MRI-based activity indices (including MAGNIFI-CD)

Several MRI-based activity indices have been developed to objectively assess disease severity in clinical trials of pfCD according to, predominantly, anatomical parameters. The Magnetic resonance Novel Index for Fistula Imaging in Crohn’s Disease (MAGNIFI-CD) has been developed to quantify fistula activity and inflammation [45] (Table 3). This index includes parameters such as fistula length, wall thickness, and the presence of abscesses, providing a standardized method to assess disease activity and response to treatment. The MAGNIFI-CD index offers a reproducible method to evaluate fistula activity, reducing interobserver variability and improving the consistency of assessments [45]. However, its sensitivity to change over short periods may be limited, affecting its responsiveness to treatment [46], similar to other radiology-based activity indices used in clinical trials, which include the Van Assche Index (VAI) [47], modified Van Assche Index (mVAI) [48], and Paediatric MRI-Based Perianal Crohn Disease (PEMPAC) Index [49]. These scoring systems are imperfect, with limitations in clinical relevance and their ability to respond to treatment, highlighting the need for a novel scoring system that can reliably detect relevant change and preferably also predict outcome [50]

**Table 3:**
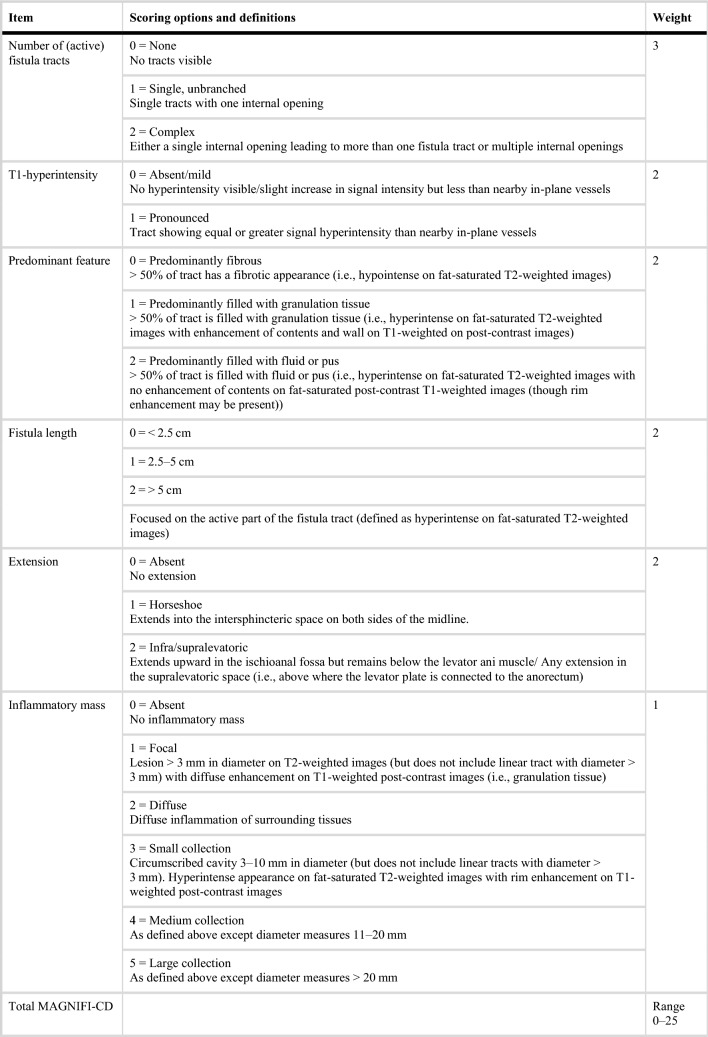
MAGNIFI-CD index (adapted from Beek et al., 2024 [44])

A survey of expert gastrointestinal radiologists in the TOpClass consortium has shown that MRI indices are not routinely used in clinical practice; contributing factors include the time-consuming nature of scoring, contrast admission, and complexity. Of clinicians surveyed, 50% think current indices are useful in a research environment, while the remaining respondents did not think MRI-based indices are useful and would like modification or new scoring systems, suggesting that future indices should be simpler and reflect changes in clinical progression if they are to be used in clinical practice.

An ideal radiological scoring index for perianal fistulizing Crohn’s disease should score highly in all domains assessed by the Consensus-based Standards for the selection of health Measurement Instruments (COSMIN) checklist of methodological quality and risk of bias [51, 52], in addition to possessing the following properties:

1. Accurately assess healing of fistulas
2. Appreciate rationalization (downstaging a complex fistula that is not amenable to repair to one that is) of complex fistulas
3. Appreciate improvement in the absence of rationalization
4. Predict response to treatment and clinical outcomes (positive and negative)
5. Demonstrate utility in clinical practice

Existing indices in pfCD may possess one or two of these characteristics, but certainly not all five. Heterogeneity and lack of clarity on the precise features of healing on MRI have made it difficult to create the perfect scoring system. Fistula MRI protocols are not standardized, and the use of contrast is optional in many centers, limiting the utility of contrast-dependent scoring systems. While MRI is a valuable tool for evaluating pfCD, existing MRI-based activity indices encounter several limitations that impede their accuracy and clinical utility.

### Volume assessment

**Fig. 5:**
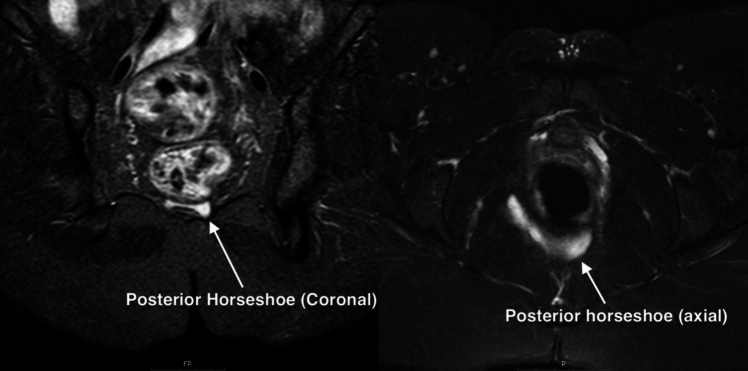
Posterior horseshoe sepsis in perianal fistulizing Crohn’s disease. Fig. 6 3D reconstruction of posterior horseshoe sepsis using manual segmentation

**Fig. 6:**
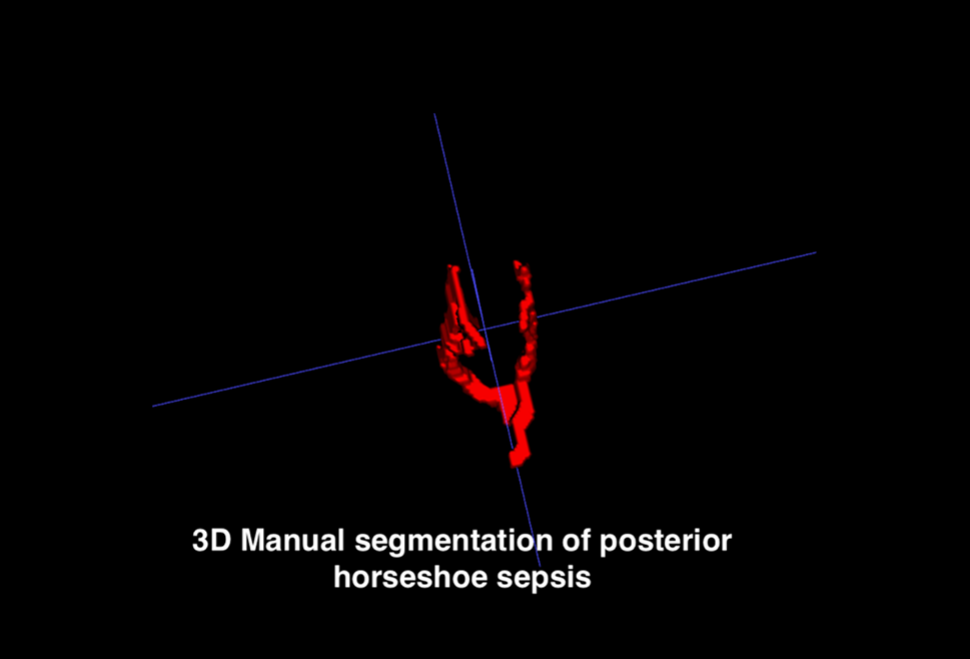
3D reconstruction of posterior horseshoe sepsis using manual segmentation

Advanced MRI techniques now allow for precise volume assessment of fistula and abscesses. Volumetric analysis of fistulae on MRI, performed by expert GI radiologists, can provide a more comprehensive understanding of disease burden and treatment effects compared with traditional, linear measurements [53, 54]. Emerging evidence suggests that calculating the volume of fistulae on MRI using manual segmentation methods can better predict clinical severity and patient symptoms and improve monitoring of treatment response by quantifying changes in fistula volume over time. This technique requires advanced imaging software and expertise, which may not be widely available, and the interpretation of volumetric data can be complex and may vary between practitioners. Further work is required before this is incorporated into routine radiological assessment methods, but this represents a promising area of research (Fig. 5 and Fig. 6)

### DCE MRI

The impact of dynamic contrast-enhanced (DCE) MRI with gadolinium has been evaluated in pfCD, with mixed results [55]. Although dynamic contrast enhancement with quantification has been suggested as a method to further refine diagnosis, it has not yet seen widespread use [42]. Additionally, quantitative MRI techniques such as diffusion-weighted imaging (DWI), DCE, magnetization transfer (MT), and T2 relaxometry hold promise for enhancing diagnostic accuracy. Specifically, DCE and DWI have demonstrated potential in assessing disease activity in pfCD, while MT might be effective in differentiating between inflammatory and fibrotic fistulae [56]. These novel techniques are not yet widely used in clinical practice or research, and further studies are needed before they are implemented routinely

### EAUS

Endoanal ultrasound (EAUS), including its 3D variant, is a valuable tool for diagnosing pfCD, particularly when MRI is not available or is unsuitable, or additional detail, particularly regarding the sphincter muscles, is needed. ESGAR guidelines suggest using EAUS to assess internal openings and the sphincter complex owing to its superior spatial resolution compared with MRI, which aids in surgical planning [34]. EAUS can provide high-resolution 2D or 3D images, with hydrogen peroxide infusion enhancing visualization by making fistula tracts more distinct [57, 58]. High-resolution 3D EAUS can reveal the Crohn’s ultrasound fistula sign (CUFS), characterized by a hypoechogenic tract surrounded by a hyperechogenic zone, which helps differentiate Crohn’s fistulae from other types [59]. Despite its utility, EAUS shows variability in diagnostic accuracy, with a reported sensitivity of 0.87 and specificity of 0.43 [60]. The presence of the CUFS has a positive predictive value of 87% and a negative predictive value of 93% for Crohn’s disease [59]. Moreover, EAUS results can influence treatment decisions, with imaging guiding therapy in 86% of cases [61]. This highlights EAUS as an important modality in the assessment and management of perianal fistulae. However, its utility is substantially limited by user experience in both scanning and image interpretation, and perhaps by the discomfort of EAUS in an acutely inflamed anus.

In the diagnosis of perianal fistula, both MRI and endoanal ultrasound (EAUS) are valuable, though each has its own strengths and limitations. However, MRI is the more commonly used imaging modality. A meta-analysis has suggested that MRI and EAUS have similar sensitivities for assessing perianal fistulae, both around 87%, though MRI generally shows higher specificity (69%) compared with EAUS (43%) [60] and is better in more complex tracts, as the resolution of EAUS diminishes further from the probe. EAUS is particularly effective in detecting intersphincteric fistulae and can be a good alternative when rectal stenosis is not a concern [23]. However, the utility of EAUS is limited by its narrow field of view; if a fistula extends beyond this range, it may remain undetected, especially in cases of ischioanal fossa or supralevator extensions, which are prone to being missed. MRI, on the other hand, excels in identifying suprasphincteric and extrasphincteric fistulae, demonstrating higher accuracy in these areas [62].

### TPUS

TPUS is particularly useful for evaluating anovaginal and rectovaginal fistulae and superficial lesions, making it a valuable complementary technique alongside MRI, especially when rectal stenosis or pain precludes EAUS. It is far less commonly used than MRI or traditional EAUS methods. While EAUS and TPUS can be effective, their performance may be limited in less experienced hands [63]. TPUS has demonstrated high sensitivity and positive predictive value for detecting and classifying perianal fistulae, validating earlier findings from studies using MRI or EAUS [63]. However, its sensitivity for identifying extrasphincteric and suprasphincteric tracts is relatively lower, likely owing to the difficulty in assessing deeper lesions that may fall outside the high-frequency ultrasound’s field of view [63].

### CT and fistulography

Fistulography and computerized tomography (CT) are not recommended for the routine diagnosis and classification of pfCD, however they may play a useful role in patients who are claustrophobic and therefore unable to tolerate MRI. CT scans can be valuable in acute settings for detecting perianal abscesses, especially when Crohn’s disease is not initially suspected. Their primary limitations include the exposure to ionizing radiation and inadequate resolution for distinguishing between fistulae and pelvic floor muscles [35].

## Innovations in imaging and biomarkers

### 3D modelling and printing

**Fig. 7:**
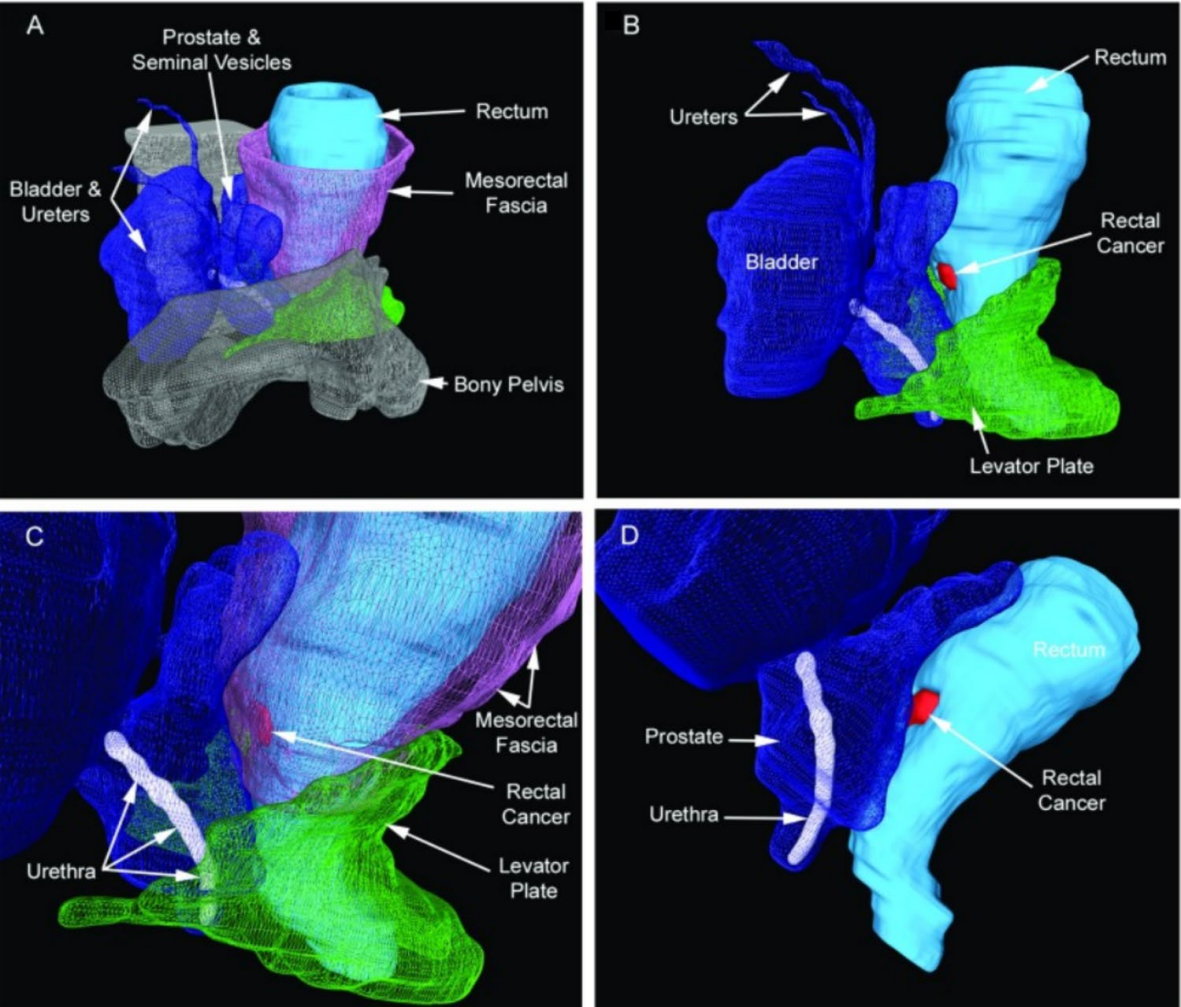
3D reconstruction for operative planning in rectal cancer [68] (*Techniques in Coloproctology*)

There has been a rapid expansion in the last decade in 3D imaging and printing. The incorporation of this emerging technology in pfCD offers significant benefits across various aspects of surgical care. The use of 3D printed models, derived from MRI data, allows for a rotatable and highly detailed visualization of the fistula’s anatomy, which enhances the precision of preoperative planning [64]. Surgeons benefit from these models by gaining clearer insights into the fistula’s complexity, including secondary tracts and deep abscesses that might be difficult to identify from MRI alone [65]. For trainees and trainers, 3D models facilitate better understanding and training, providing a tangible reference that improves educational outcomes and surgical technique. Patients also gain from this technology, as 3D reconstructions can enhance communication about their condition and the planned surgical approach, thereby improving their overall experience and understanding of the procedure [66, 67]. The utility of this technology is constrained by its high costs, lengthy processing times (taking days to produce just a few models), and the lack of automation. As a result, creating a 3D model requires time-consuming manual segmentation of fistulae by an expert radiologist. In the near future, volume assessment, measurement, and production of a 3D model (virtual or physical) will be automated (Fig. 7).

### Radiomics

Radiomics, the advanced analysis of medical imaging data to extract quantitative features, is increasingly being explored in the context of pfCD and luminal Crohn’s disease [69]. In pfCD, radiomics can offer detailed insights into the disease’s complexity, helping to characterize fistulae and abscesses more precisely than conventional imaging. This approach leverages features from imaging studies, such as MRI or CT scans, to predict disease progression and treatment outcomes. For instance, research by Fiorino et al. demonstrated that assessing bowel damage in CD using the Lémann index on cross-sectional imaging was a strong predictor of future intestinal resection and related hospitalizations [70, 71]. Similarly, in luminal CD, radiomics can enhance understanding of disease activity and severity by analyzing imaging data to identify patterns associated with clinical outcomes. This emerging technology promises to improve personalized treatment plans and prognostic accuracy by integrating quantitative imaging features into clinical decision-making. However, there have been no large-scale real-world effectiveness studies on the use of radiomics in the field of pfCD to date, and this is an area for future research.

### Artificial intelligence (AI) and machine learning (ML)

AI and ML algorithms are exciting developments that are being increasingly applied to medical imaging for pfCD [72]. These technologies can analyze large datasets to detect subtle changes and patterns, improve diagnostic accuracy, and predict treatment outcomes [73]. AI offers the potential to improve the accuracy and efficiency of pfCD diagnosis on MRI, reducing the workload on radiologists and providing consistent, high-quality assessments. However, similar to the integration of radiomics into pfCD research, implementing AI in clinical practice demands significant investment in technology, training, and the creation of complex neural networks. These networks require large datasets to generate meaningful outcomes, presenting challenges in both resource allocation and data availability. Additionally, the “black box” nature of many AI algorithms presents challenges for interpretability and clinical trust. The application of these emerging techniques in inflammatory bowel disease, particularly in perianal Crohn’s disease, remains in its early stages, and further research is required before we are likely to see clinically relevant models.

### Biomarkers

In diagnosing and managing pfCD, biomarkers such as C-reactive protein (CRP) and fecal calprotectin (FC) can play a role in assessing disease activity and guiding treatment. CRP, an acute-phase reactant, reflects systemic inflammation but may not always correlate with disease severity in CD [74]. It can be used to guide the presence of perianal abscess in the clinic, especially in patients with supralevator sepsis or complex pfCD that may not be apparent on clinical examination alone. FC, a protein found in granulocytes as they infiltrate the gastrointestinal tract [75], is a more specific marker of intestinal inflammation and has demonstrated high sensitivity (0.95) and specificity (0.91) for diagnosing inflammatory bowel disease [76]. Ongoing research has suggested that FC can be used to differentiate between pfCD and cryptoglandular disease, with higher levels associated with more complex pfCD [77]. Further research by the same group indicates that, in CD with active perianal fistulas, elevated FC levels do not reliably differentiate between patients with mucosal ulcers and those with endoscopically inactive disease [78]. A biomarker that is specific for pfCD and that can predict disease onset while also correlating with disease activity and severity is an unmet research need and will revolutionize the management of pfCD, in particular for those patients with pfCD who are currently treated without Crohn’s medications because of a lack of luminal or histologic diagnosis.

### Isolated perianal Crohn’s disease (ipCD)

Crohn’s disease is identified by diagnostic imaging and histological features, usually in the bowel lumen, but diagnostic histological features can also be determined in perianal fistulas. Histologically proven ipCD is a recognized phenomenon, but since histological evidence of CD may be found in as few as 10% of perianal fistula tract biopsies in patients with known CD, and since ipCD exists as an entity, it follows that some patients with perianal Crohn’s disease will have neither evidence of luminal disease nor histological features in the fistula to confirm the diagnosis.

**Fig. 8:**
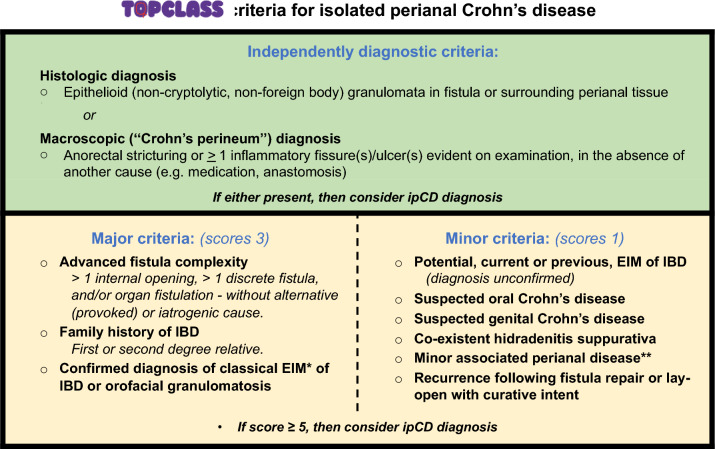
The novel ipCD scoring system. *EIM = extra-intestinal manifestation. **Minor associated perianal disease is defined by a single large (> 1 cm) or oedematous skin tag; multiple small tags [3+]; non-fistulising perianal skin inflammation; or natal cleft ulceration

This population is difficult to distinguish objectively from cryptoglandular anal fistula. Therefore, the TOpClass consortium has developed recommendations on the investigation and diagnosis of patients with suspected but unproven ipCD, and also in whom and how to consider CD treatment (Fig. 8). This will be useful both clinically and in trials that wish to identify or exclude such patients from their cohorts.

## Conclusions

The diagnosis of pfCD has evolved considerably, with advancements in imaging technologies, biomarkers, and clinical assessment tools enhancing diagnostic accuracy and patient care. MRI, including noncontrast and contrast-enhanced techniques, MRI-based activity indices, volumetric assessment, radiomics, and AI, have or may revolutionize imaging in pfCD [45, 53, 66, 73, 79]. Novel biomarkers, molecular diagnostics, single-cell sequencing, and genomics offer promising avenues for noninvasive and accurate disease monitoring [80–82]. Additionally, standardized clinical assessment tools, despite their limitations, contribute to consistent and comprehensive evaluation of disease severity and treatment outcomes [83]. The development of patient-reported outcome measures (PROMs) and the integration of patient-directed approaches underscore the importance of patient-centred care in pfCD management [84]. These innovations collectively improve the diagnostic landscape of pfCD, fostering better management strategies and ultimately improving patient outcomes. Continued research and integration of these advancements into clinical practice are essential for further progress in the diagnosis and management of pfCD.

## Data Availability

No datasets were generated or analyzed during the current study.
